# Food-Grade Titanium Dioxide Induces Toxicity in the Nematode *Caenorhabditis elegans* and Acute Hepatic and Pulmonary Responses in Mice

**DOI:** 10.3390/nano12101669

**Published:** 2022-05-13

**Authors:** Giovanni Sitia, Fabio Fiordaliso, Martina B. Violatto, Jennifer Fernandez Alarcon, Laura Talamini, Alessandro Corbelli, Lorena Maria Ferreira, Ngoc Lan Tran, Indranath Chakraborty, Mario Salmona, Wolfgang J. Parak, Luisa Diomede, Paolo Bigini

**Affiliations:** 1 Experimental Hepatology Unit, Division of Immunology, Transplantation and Infectious Diseases, IRCCS San Raffaele Scientific Institute, Via Olgettina 58, 20132 Milano, Italy; sitia.giovanni@hsr.it (G.S.); ferreira.lorena@hsr.it (L.M.F.); tran.ngoclan@hrs.it (N.L.T.); 2 Department of Molecular Biochemistry and Pharmacology, Istituto di Ricerche Farmacologiche Mario Negri IRCCS, Via Mario Negri 2, 20156 Milano, Italy; fabio.fiordaliso@marionegri.it (F.F.); martina.violatto@marionegri.it (M.B.V.); jennifer.fernandez_alarcon@marionegri.it (J.F.A.); laura.talamini@marionegri.it (L.T.); alessandro.corbelli@marionegri.it (A.C.); mario.salmona@marionegri.it (M.S.); 3 Center for Hybrid Nanostructures (CHyN), Hamburg University, Luruper Chaussee 149, 22607 Hamburg, Germany; indranath@iitmandi.ac.in (I.C.); p1pv041@uni-hamburg.de (W.J.P.)

**Keywords:** titanium dioxide, E171, nanoparticles, *C. elegans*, mice, toxicity

## Abstract

Food-grade titanium dioxide (E171) contains variable percentages of titanium dioxide (TiO_2_) nanoparticles (NPs), posing concerns for its potential effects on human and animal health. Despite many studies, the actual relationship between the physicochemical properties of E171 NPs and their interaction with biological targets is still far from clear. We evaluated the impact of acute E171 administration on invertebrate and vertebrate animals. In the nematode, *Caenorhabditis elegans*, the administration of up to 1.0 mg/mL of E171 did not affect the worm’s viability and lifespan, but significantly impaired its pharyngeal function, reproduction, and development. We also investigated whether the intravenous administration of E171 in mice (at the dose of 6 mg/kg/body weight) could result in an acute over-absorption of filter organs. A significant increase of hepatic titanium concentration and the formation of microgranulomas were observed. Interstitial inflammation and parenchymal modification were found in the lungs, coupled with titanium accumulation. This was probably due to the propensity of TiO_2_ NPs to agglomerate, as demonstrated by transmission electron microscopy experiments showing that the incubation of E171 with serum promoted the formation of compact clusters. Overall, these data emphasize the actual risk for human and animal exposure to E171.

## 1. Introduction

Understanding the biological interactions between nanoparticles (NPs)—generated in large-scale production processes—and biological matrices (i.e., cells, tissues, and whole organisms) is important for defining their impacts on human and environmental health [1]. Particular concern is raised by the evidence that NPs of different origins and chemical compositions might have negative effects, such as the production of radical oxygen species (ROS) and the activation of inflammatory responses in peripheral organs of the digestive and respiratory systems [2,3,4]. 

Out of the wide range of nanomaterials—produced either on purpose or unintentionally in the industrial process—titanium dioxide (TiO_2_) is the most globally used, with 3000 tons produced each year and a diverse range of applications [5,6]. TiO_2_ is chemically stable and difficult to dissolve in aqueous solutions [7]. Its exposure in workplaces raises significant questions about the occupational risks for workers, because, as stated by the International Agency for Research on Cancer (IARC) [8], TiO_2_ bulk material is a possible carcinogen for humans if inhaled, and is classified as 2B [9]. 

Occupational exposure to applied TiO_2_ (typically nanosized) is regulated by two additional regulatory guidelines: the National Institute for Occupational Safety and Health (NIOSH; Washington D.C., USA) [10] and expert panels from the New Energy and Industrial Technology Development Organization (NEDO; Tokyo, Japan) [11]. The inertness and persistence of TiO_2_ NPs in the body might be a critical issue in terms of safety and large-scale production plans. For that reason, closer attention must be paid to its exposure.

On account of their physicochemical properties, TiO_2_ NPs are used for a variety of applications, such as in the manufacturing of high refractive index products (e.g., coatings, plastics, and paints), photocatalysts (water treatment and air purification), agriculture (fertilizers and pesticides) [12], food industry (processing and packing of food), and in pharmaceutical industries (cosmetics and toothpastes) [6,7,8,9,10,11,12,13,14,15]. 

In nature, TiO_2_ primarily occurs in the form of minerals such as brookite, rutile, and anatase. However, only rutile and anatase can be used without surface treatment and coating. Furthermore, they have a high number of industrial applications as the food additive, E171 [16,17]. However, anatase is 100 times more toxic than rutile [18]. E171 consists of particles typically ranging from 20–300 nm in diameter [15], and the percentage of NPs with a diameter of < 100 nm ranges from 10% to 45%, depending on their manufacturing conditions [18,19,20,21]. According to European Union Regulations 2011/696/EU [22], E171 cannot be considered a nanomaterial because the number of NPs with a size range below 100 nm is less than 50%. However, the wide use of E171 in the daily products ingested by millions of consumers, as well as its possible dispersion into the environment, have raised awareness of its potential side effects on human and animal health [23].

Once ingested, TiO_2_ NPs can cross biological membranes, enter cells, and accumulate in tissues and organs, where they exert toxicity and can trigger both local and systemic responses [24]. In addition, once it enters the circulation, E171 perfuses all peripheral organs (including the lungs and liver), accumulating in the digestive organs and impairing gastrointestinal functions [25]. It can also accumulate in the reproductive system, pass through the placenta, and be transferred from maternal to fetal circulation [24,25,26,27,28].

Despite a large number of studies, the actual relationship and interactions between the physicochemical properties of food-grade TiO_2_ NPs and their biological targets, are still far from clear. Although the results of in silico and in vitro analyses are absolutely necessary to have even the first idea of the potential toxicity of NPs on the human body—such as an alteration in cell cycles, constriction of nuclear membranes, and apoptosis—they are rarely transferable to multicellular organisms [29,30,31]. The recent decision from the EU to ban E171 as a food additive [16] strengthens the potential risks even more, including the acute toxicity related to its over-exposure. Thus, testing the toxicity of E171 in cells up to vertebrates, at increasing levels of biological complexity, is urgently needed. 

In the present study, we evaluated the acute toxicity of E171 in two different animal models: the invertebrate nematode, *Caenorhabditis elegans* (*C. elegans*), and mice. The E171 administered in this work has been deeply characterized in our previous studies through single-particle inductively coupled plasma-mass (spICP-MS), dynamic laser light scattering (DLS), nanoparticle tracking analysis (NTA), and transmission electron microscopy (TEM) [32]. A mean particle diameter of 201.2 ± 8.5 nm and 326.4 ± 9.2 nm were measured by NTA and DLS, respectively, with a polydispersity index of 0.18 ± 0.02. With TEM, we found that 35% of these particles had a diameter below 100 nm, which was confirmed using ICP-MS [32]. We evaluated the effects of E171 on an invertebrate nematode (*C. elegans*), which had already employed to test the effects of TiO_2_ NPs of various shapes and sizes [33,34,35,36]. Previous studies show that rod-shaped TiO_2_ NPs are more toxic for worms than bipyramidal and spherical ones, severely affecting pharyngeal function, reproduction, and larval growth—although no differences in biological distribution and accumulation have been found [34]. E171, at doses that did not affect the worm’s viability or lifespan, were found to significantly impair pharyngeal function, reproduction, and development. It is important to note that the toxicity data on worms provide information from a whole-animal perspective, with intact and metabolically active digestive, reproductive, endocrine, sensory, and neuromuscular systems [35,36]. This nematode is a reliable model for human pathophysiology because it exhibits similar toxic modes of action to mammals [37]. *C. elegans* was selected not only for its well-conserved signal pathways and its high level of gene homology with humans—but because it can also be used to obtain useful information regarding the toxicity of E171 for environmental health (particularly for terrestrial and aquatic animals), as this nematode is, in fact, ubiquitous worldwide, and can live in both soil and water [38]. 

We recently discovered that repeated oral administration of E171 to NFR mice, at a dose level comparable to that of the estimated human dietary exposure, resulted in titanium (Ti) deposition in the digestive system, massive intestinal adsorption, bloodstream circulation, and inflammatory action in the liver [32]. In the present study, we focused our attention on the fate of E171 after its penetration into the bloodstream, mimicking over-exposure in humans, as TiO_2_ levels found in food and personal care products range from 0.5 to 9 mg/kg [19,39]. The absorption rate of TiO_2_ NPs is incredibly low, so in order to avoid any confounding factors due to upstream sources of stress (e.g., cytokine activation due to gastrointestinal injury) and mimic the potential risk of over-exposure to E171, we chose a single intravenous administration at a dose of 6 mg/kg body weight (b.w.), which would be administered to healthy immunocompetent mice. Although toxic effects of E171 have been reported after systemic administration [40], to date, little information is available on the impact of E171 on filtering organs after long-term exposure. In this study, we focused our attention on how food-grade TiO_2_ is distributed, how it is transported through the bloodstream, and whether it is capable of inducing pathological changes in the major filtering organs (i.e., lungs and liver). Moreover, to determine whether the serum affects the colloidal stability of E171, inducing possible structural modifications and/or the formation of aggregates once in circulation, transmission electron microscopy (TEM) analysis was performed. This control is crucial for correlating the physicochemical properties of E171 particles with their behavior in biological fluids. We also studied the biodistribution of E171 in different organs at different time points after administration (from one hour to one week) and evaluated a number of biochemical and histological parameters that are indicative of toxic profile-related alterations. 

The obtained results showed that E171 accumulated in the livers of the treated mice, where it formed aggregates and hepatic inflammatory microgranulomas. Progressive accumulation was also detected in the lungs and was accompanied by massive recruitment of monocytes, macrophage hypertrophy, and focal parenchymal lesions in the alveoli. These data provide an interesting overview of the impact of E171 on human and animal health.

## 2. Materials and Methods

### 2.1. C. elegans Studies

The *C. elegans* strain, Bristol N2, was obtained from the *Caenorhabditis elegans* Genetic Center (CGC; University of Minnesota). It was propagated at 20 °C on a solid nematode growth medium (NGM) seeded with *Escherichia coli* OP50 (from CGC) as food. The effects of E171 on worm mortality, pharyngeal function, reproduction, and development were investigated, as per the method described by Iannarelli et al. (2016) [34]. 

To determine the impact on pharyngeal function, age-synchronized nematodes (L4 larval stage) were collected with M9 buffer, centrifuged, and washed twice with 5 mM phosphate-buffered saline (PBS) (pH 7.4) to remove bacteria. Worms were suspended in water and incubated with freshly suspended E171—without *E. coli*, to avoid potential interference between bacteria and the nanomaterial—at a concentration of 0.01–1.0 mg/mL (100 worms/100 μL). Control worms were incubated only with water (100 worms/100 μL). After 2 h, the worms were transferred to NGM plates seeded with OP50 *E. coli*. Mortality was recorded 2 h and 24 h after treatment by counting the paralyzed worms. At these same times, pharyngeal pumping efficiency was measured by counting the number of times the terminal bulb of the pharynx contracted during a 1-min interval (pumps/min).

Age-synchronized L4 nematodes were used to determine the effects of E171 on reproduction and larval development. Worms were incubated for 2 h without *E. coli,* with only freshly suspended E171 in water, at a concentration of 0.1–0.5 mg/mL (100 worms/100 μL). Control worms were incubated only with water (vehicle). *C. elegans* were then transferred to NGM plates seeded with fresh OP50 *E. coli*, and the eggs laid were counted after 6 h of incubation at 20 °C. The adults were then removed, and the plates were stored at 20 °C. The worms in the different larval stages (L1, L2, L3, L4, and adult) were counted 16, 24, 36, and 48 h after the eggs hatched.

For lifespan experiments, worms at the L3 larval stage were fed for 2 h with either 0.2 mg/mL of E171 freshly suspended in water (100 worms/100 µL) or water alone (vehicle). Nematodes were then transferred to fresh NGM plates seeded with *E. coli*. To avoid overlapping generations, worms were transferred to fresh NGM plates seeded with *E. coli* every day until they stopped laying eggs. The live worms were counted on each consecutive day until all worms were dead, and the number of live worms was scored.

### 2.2. Transmission Electron Microscopy (TEM)

Food-grade TiO_2_ (E171, Pretiox AV01PhG) was kindly provided by Giusto Faravelli S.p.A. (Milan, Italy). The product was 99.3% pure TiO_2_ anatase, complying with the standard regulations of the European and US Pharmacopoeia, the Food and Drug Administration (FDA), and the Food Additive Regulations. To investigate whether serum could affect the morphology and colloidal properties of E171 over time, TEM analysis was performed, incubating 1 mg/100 µL of E171 in either H_2_O or murine serum for 1, 4, or 24 h. To avoid any effects on the particle characteristics that would not occur in realistic conditions of use, the samples were not sonicated. From each sample, 5 μL were extracted, placed into a formvar/carbon-coated copper grid (100 mesh) (Electron Microscopy Sciences, Washington, PA, USA), and air-dried at room temperature. NP images were obtained with an energy-filtered transmission electron microscope (EFTEM, ZEISS LIBRA^®^ 120, Milano, Italy) coupled with an yttrium aluminum garnet (YAG) scintillator slow-scan CCD camera (Sharp eye, TRS, Moorenweis, Germany) with a focus aid option, enabling us to reach the precise eucentric plane in the observation of each single NP [21]. 

### 2.3. CD1 Mouse Studies

The Mario Negri Institute for Pharmacological Research IRCCS adheres to the principles set forth in the following laws, regulations, and directives concerning the care and use of laboratory animals: Italian law (D.lgs 26/2014; Authorization No. 19/2008-A issued on March 6, 2008 by the Ministry of Health); the Mario Negri Institutional Regulations and Policies—which provide internal authorization for persons conducting animal experiments (Quality Management System Certificate, UNI EN ISO 9001:2015, Reg. No. 6121); the NIH Guide for the Care and Use of Laboratory Animals (2011 edition); and the EU Directives and Guidelines (EEC Council Directive 2010/63/UE). This work was reviewed by the IRCCS-IRFMN Animal Care and Use Committee (IACUC) and subsequently approved by the Italian “Istituto Superiore di Sanità” (Code: 42/2016- PR).

Eight-week-old male CD1 mice (Charles River, Italy) were housed in specific pathogen-free animal rooms, with a constant temperature of 21 ± 1 °C, a humidity of 55 ± 10%, a 12-h light–dark cycle, and free access to food and water. Mice were randomly divided into two groups (9/group). One group was injected intravenously with 6 mg/kg b.w. of E171 suspended in 200 μL of injection-grade distilled water. The control group received only 200 μL of injection-grade distilled water (vehicle). At selected times (before injection and 1, 12, 24, 96, and 168 h after), mice were anesthetized with 5% isoflurane, and their blood was collected in heparinized tubes from the retro-orbital plexus for analysis, as described previously [41]. Blood from vehicle-treated mice was also used to prepare serum to check the colloidal stability of E171 by TEM. Then, at 1, 24, and 168 h after E171 administration, mice were euthanized (3 animals per time point). Liver, kidneys, spleen, brain, and lungs were collected and cleaned with ultrapure water. Subsamples were then collected to determine TiO_2_ content by inductively coupled plasma-mass spectrometry (ICP-MS), and a histological examination was conducted on the livers and lungs. 

### 2.4. Biodistribution of TiO_2_

TiO_2_ levels in the explanted liver, spleen, kidneys, lungs, brain, and blood were quantified using ICP-MS (Agilent 7700 series, Santa Clara, CA, USA) [42]. The mouse samples were first digested by the addition of 3 mL of ultra-pure HNO_3_ (67 wt %) (Fisher Chemical). Samples were constantly agitated in 50 mL Falcon tubes for 72 h at 22 °C until the solution became clear and no organics were left in the tube. From this mixture, 100 µL were taken and further digested with 100 µL of HF acid for 48 h to ensure the atomic incidence of the former TiO_2_ NPs. The Ti concentration given by ICP-MS in the original HNO_3_ solution is C_Ti_ = C′_Ti_ α_dil_ (C′ is the actual ICP-MS reading and α_dil_ is the dilution factor). The total mass of Ti in each organ is m_Ti_ [g] = C_Ti_ V_HNO_3__. We calculated the %ID as m_Ti_ (in each organ)/M_Ti_ (injected Ti mass) * 100.

### 2.5. Blood Analysis 

Complete blood cell counts were taken from fresh whole blood collected in EDTA-coated microvettes (Sarstedt, Numbrecht, Germany) using an automated cell counter (IDEXX Procyte Dx, IDEXX Laboratories, Hoofddorp, Netherland). The extent of hepatocellular injury and whole-body toxicity was recorded by measuring serum alanine aminotransferase (sALT), serum aspartate aminotransferase (sAST), and lactate dehydrogenase (LDH) activity. A kinetic UV method, optimized by the International Federation of Clinical Chemistry and Laboratory Medicine, was used in an Aries chemical analyzer (Werfen Instrumentation Laboratory S.p.A., Milano, Italy). Values were expressed as Units/Liter (U/L). Each analysis was validated by a certified biochemical chemistry and hematology specialist using quality-controlled blood (CQI) at the San Raffaele Mouse Clinic (http://research.hsr.it/en/services/mouse-clinic/hematologic-testing.html, accessed on 21 October 2021).

### 2.6. Histopathology

Organs were harvested, fixed in zinc formalin, processed, embedded in paraffin, sectioned, and either stained with hematoxylin–eosin (HE) or were further processed for immunohistochemical analysis, as previously described [41]. Immunohistochemical staining for tissue-resident monocytes/macrophages was conducted with the anti-F4/80 specific antibody (clone A3-1, AbD Serotec, Luxembourg, Luxembourg). All images were acquired using the Aperio Scanscope CS2 system (Leica Biosystems, Milano, Italy), available at the San Raffaele Advanced Light and Electron Microscopy BioImaging Center (ALEMBIC, Milano, Italy). Images were identified as representative areas of interest within the total area of the specimen, then analyzed and exported as ImageScope snapshots.

### 2.7. Statistical Analysis

Data were analyzed using GraphPad Prism 8.0 software. Differences between data groups were analyzed as follows. An independent Student’s *t*-test was used for larval growth and F4/80 quantification in the lungs. One-way ANOVA, followed by a Bonferroni post hoc test, were used to analyze the effects on pharyngeal pumping rate, the number of eggs laid in the *C. elegans* studies, and the Ti accumulation measured by ICP-MS. Two-way ANOVA, followed by a Bonferroni post hoc test, were used to analyze the serum markers for toxicity (ALT, AST, and LDH). The Gehan–Breslow–Wilcoxon test was used to assess the survival rate of *C. elegans*, and a two-tailed Mann–Whitney test was conducted on the blood cell counts. A *p* value of < 0.05 was considered statistically significant and was reported on the graphs. Data are presented as either the mean ± standard deviation (SD) or the mean ± standard error of the mean (SEM) and are depicted in each figure legend. 

## 3. Results 

### 3.1. Toxicity of E171 in C. elegans 

**Figure 1:**
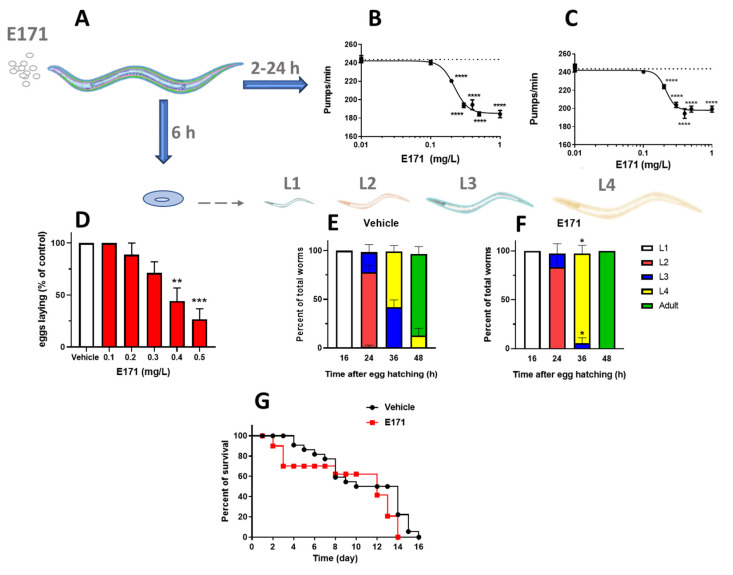
Effects of E171 in *C. elegans*. (**A**) Worms (100 worms/100 µL) were fed for 2 h with E171 freshly suspended in water in the absence of OP50 *E. coli*, then plated on NGM plates seeded with the bacteria. Behavioural studies were conducted at different times after plating. Control worms were fed only water. (**B**,**C**) The effect of 0.01–1 mg/L E171 on the pharyngeal pumping rate was scored (**B**) 2 h and (**C**) 24 h after plating (vehicle, dotted line). Data are given as the mean ± SEM (30 worms per group); **** *p* < 0.01 vs. vehicle, according to one-way ANOVA and Bonferroni post hoc tests. (**D**) The number of eggs laid 6 h after plating is expressed as a percentage of the eggs laid by vehicle-fed worms. Mean ± SEM (10 worms per group); ** *p* < 0.005 and *** *p* < 0.0005 vs. vehicle, according to one-way ANOVA and Bonferroni post hoc tests. (**E**,**F**) Larval growth was rated 16, 24, 36, and 48 h after eggs hatched by counting the worms at different larval stages. Data are given as percentages of the total worms ± SEM; * *p* < 0.05 vs. vehicle at the same time point, according to Student’s *t*-test. (**G**) Survival rate after injection of 0.2 mg/L E171. Median survival of vehicle = 14 days; E171 = 12 days. Curve comparison: *p* = 0.487 log-rank analysis and *p* = 0.282, according to the Gehan–Breslow–Wilcoxon test.

The toxicity of E171 in *C. elegans* was evaluated by analyzing different behavioral endpoints (Figure 1A–G). Worms were given E171 at subtoxic doses (0.01–1 mg/mL), which did not cause any significant increase in mortality after either 2 or 24 h (data not shown). In this concentration range, dose-dependent inhibition of the nematodes′ pharyngeal function was observed 2 h after feeding, with a 50% inhibitory concentration (IC50) of 0.221 ± 0.10 mg/L (Figure 1B). Pharyngeal inhibition was still comparable 24 h after feeding (IC50 = 0.211 ± 0.10 mg/mL), indicating that E171 caused a lasting change in feeding (Figure 1C). The effect had already reached a plateau at 0.5 mg/L of E171, which caused 76.5% and 82.2% reductions in pharyngeal function at 2 h and 24 h after treatment, respectively.

The effect of 0.1–0.5 mg/L of E171 on the ability of adult worms to reproduce was investigated by counting the number of laid eggs (Figure 1D). E171, in doses up to 0.3 mg/L, did not affect egg laying. However, there was a significant reduction in the reproductive capacity for concentrations ranging from 0.4 to 0.5 mg/L, with levels of 56% and 73%, respectively. E171, at 0.2 mg/L, modified the larval growth of worms, causing a significant increase in the speed of development compared to the vehicle (Figure 1E,F). Thirty-six hours after eggs hatched, E171-fed worms had a higher percentage of worms in L4 (91.7 ± 8.3%) than vehicle-fed worms (57.15 ± 6.2%), resulting in a much higher percentage of adult worms 12 h later. We also examined whether 0.2 mg/L of E171 affected worm survival, but no significant alterations were found. A median survival of 14 days and 12 days were observed for vehicle-fed worms and E171-fed nematodes, respectively (Figure 1G). 

These findings indicate that E171—at doses that did not modify the viability or the lifespan of *C. elegans*—instead affected their health through the onset of sub-toxic phenotypes, such as impairments in feeding and normal development.

### 3.2. Toxicokinetic Studies in Mice

**Figure 2:**
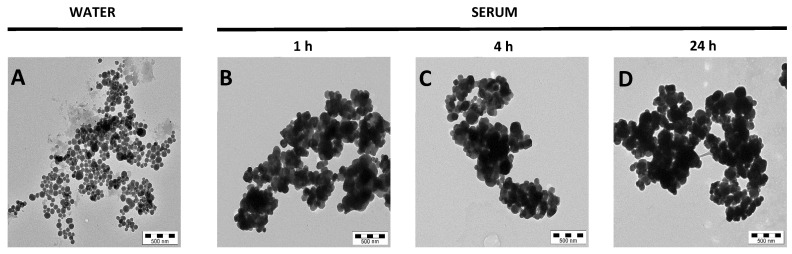
Effect of serum on E171 particles. Representative TEM images of E171 incubated in (**A**) water or (**B**–**D**) murine serum for 1, 4, and 24 h. TEM scale bars = 500 nm.

Despite the fact that ingested E171 is poorly absorbed into the blood circulation, a small percentage of TiO_2_ NPs are able to circulate in the bloodstream for a long time due to their long half-lives, ranging from 28 to 650 days [16]. To investigate whether serum affected the colloidal properties of E171, TEM analysis was conducted on the material suspended in either water or murine serum and incubated for 1, 4, and 24 h at 37 °C. At every time point, it was observed that E171 particles in the water were homogenously distributed with single-particle differentiation, making it possible to clearly distinguish different sizes of several TiO_2_ NPs (Figure 2A). However, when E171 was suspended in serum, the particles formed large compact clusters (0.5–1 µm) regardless of the incubation time, making it almost impossible to discriminate single NPs (Figure 2B–D). These findings indicate that serum affects the morphological characteristics of E171, promoting the clustering of TiO_2_ particles and influencing their biodistribution in vivo. 

**Figure 3:**
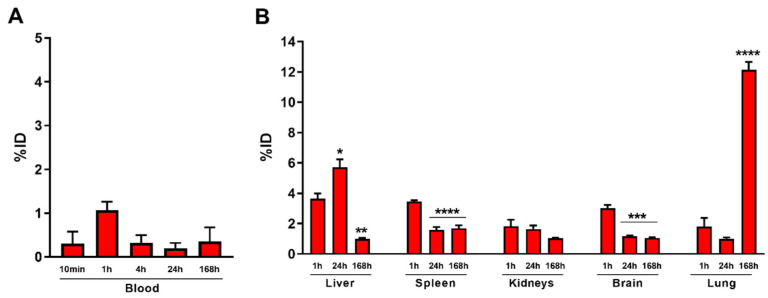
Titanium content in organs. Elemental titanium in (**A**) blood, (**B**) liver, spleen, kidneys, brain, and lungs of mice intravenously treated with 6 mg/kg b.w. E171. Ti content was determined using inductively coupled plasma mass spectrometry (ICP-MS) on tissues collected at different times after the E171 treatment. Data are given as percentages of the injected dose (% ID). Each data point represents the mean ± SEM (N = 3); * *p* < 0.05, ** *p* < 0.005, *** *p* < 0.0005, and **** *p* < 0.0001 vs. 1 h, according to one-way ANOVA and Bonferroni post hoc tests.

Healthy mice were then intravenously treated with E171 (6 mg/kg b.w.), and the elemental Ti concentrations (originating from the TiO_2_ NPs) in blood (Figure 3A) and filtering organs (Figure 3B) were determined by ICP-MS—from one hour up to one week after injection. Ti blood concentrations peaked one hour after injection, representing about 1% of the injected dose (ID). They then decreased substantially by ~60% after 4 h, and remained low for up to 168 h. This finding suggests that circulating E171 NPs were either rapidly accumulated in filtering organs or were being cleared out from the organism. The low TiO_2_ concentrations (in terms of detected Ti), as an ID percentage in liver, spleen, and kidneys, seem to confirm the hypothesis of rapid clearance and poor filtration by liver and spleen macrophages. The lack of accumulation in the kidneys at 1 and 24 h discards renal clearance as a potential explanation. As expected, there was a drastic decrease in TiO_2_ concentrations in the brain (in terms of detected Ti) between 1 and 24 h after injection. It is therefore likely that the amounts measured at the early time points were almost entirely due to the presence of TiO_2_ NPs circulating in the cerebral vessels. In contrast, the pattern of Ti accumulation in the lungs was surprisingly higher compared to other organs, and these levels increased over time (Figure 3B). 

**Figure 4:**
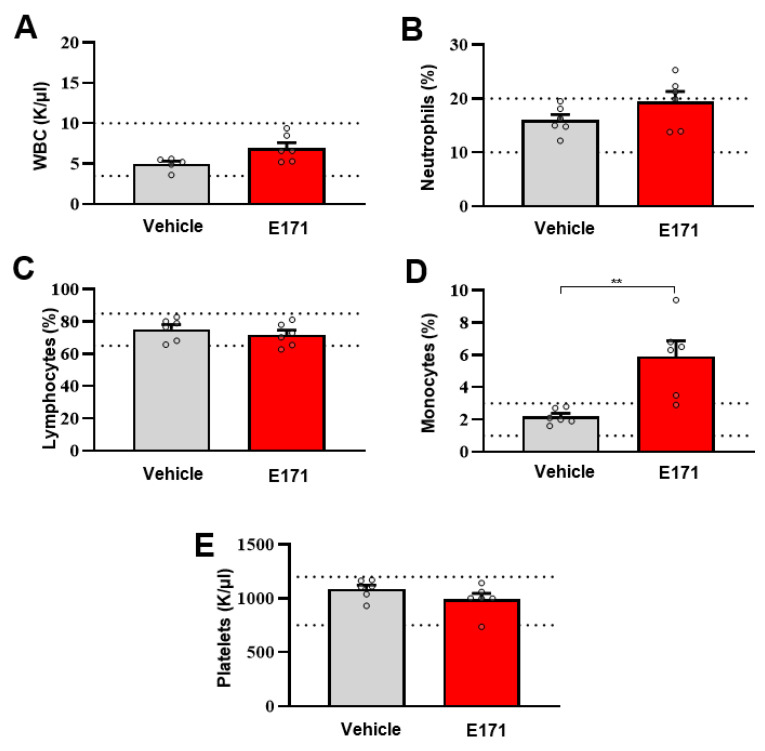
Effect of E171 on complete blood cell count. (**A**) Total white blood cells (WBC), (**B**) neutrophils, (**C**) lymphocytes, (**D**) monocytes, and (**E**) platelets of mice, measured 12 h after the intravenous injection of E171. Control mice were treated with vehicle alone (Vehicle). The dotted lines indicate the upper and lower normal values for each variable (4–10 K/μL for WBC; 65–87% for lymphocytes; 10–20% for neutrophils; 1–3% for monocytes; and 750–1250 K/uL for platelets). Data are given as mean ± SEM (N = 6); ** *p* < 0.005 vs. vehicle mice, according to a two-tailed Mann–Whitney test.

In order to define the toxicity profile of E171 on circulating hematopoietic cells, a complete blood count was obtained 12 h after either the vehicle or E171 injection. The blood cell analysis showed that the total numbers of circulating WBC, lymphocytes, and neutrophils were not affected by E171 administration (Figure 4A–C), whereas the number of monocytes increased significantly (Figure 4D). Monocytes are the circulating myeloid cells that, once recruited to tissues and organs such as the liver, differentiate into macrophages devoted to eliminating many exogenous molecules (including E171) through phagocytosis [43]. Therefore, it is possible that circulating monocytes were rapidly recruited to remove TiO_2_ particles from the bloodstream and other organs. Importantly, there were no differences in the circulating platelets (Figure 4E), suggesting that E171 did not affect platelet activation and function. 

**Figure 5:**
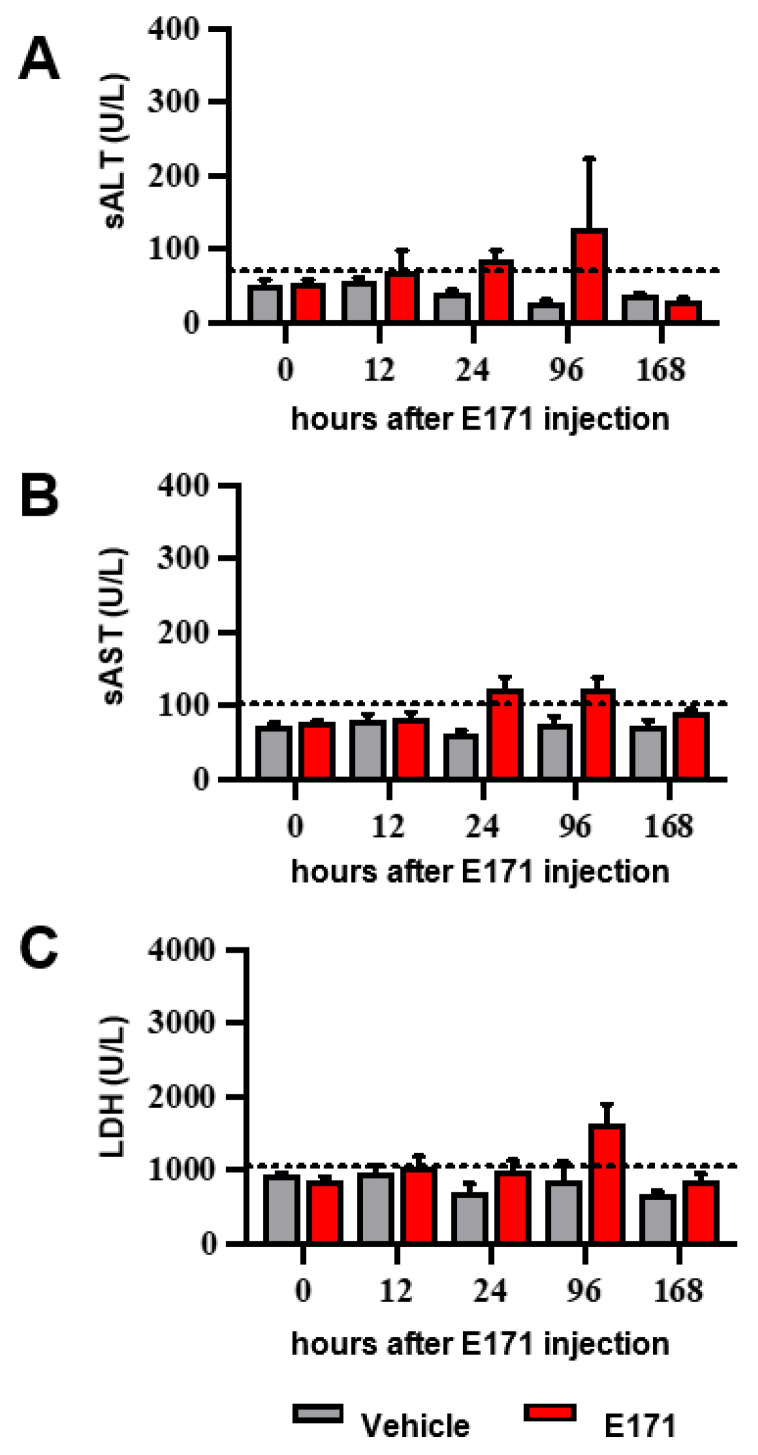
Effect of E171 on serum biochemical parameters. (**A**) sALT, (**B**) sAST, and (**C**) sLDH activity were measured in serum of mice before and after different E171 or vehicle injection times. The dotted lines indicate the upper normal value for each variable (43 U/L for sALT; 104 U/L for sAST; and 1100 U/L for LDH). Data are expressed a Units/Liter (U/L). Values are the mean ± SEM (N = 6). No significant differences were found between E171-treated mice vs. vehicle mice, according to two-way ANOVA and Bonferroni post hoc tests.

Although the levels of Ti originating from TiO_2_ NPs in blood, liver, kidneys, spleen, and brain were indicative of low accumulation, we examined whether they also potentially caused signs of toxicity. Serum biochemical levels of alanine aminotransferase (sALT) (Figure 5A), a hepatocellular enzyme released into the bloodstream after hepatocellular cell death [32], along with aspartate aminotransferase (sAST) and lactate dehydrogenase (LDH) (Figure 5B,C) released into the circulation after cell death in the liver and other tissues (i.e., blood cells, kidneys, brain, and lungs) [44], were measured as markers of tissue damage. No significant differences in the levels of sALT, sAST, and LDH were observed in animals treated with E171 compared to vehicle-treated mice, indicating that Ti accumulation in organs did not induce overt toxicity.

**Figure 6:**
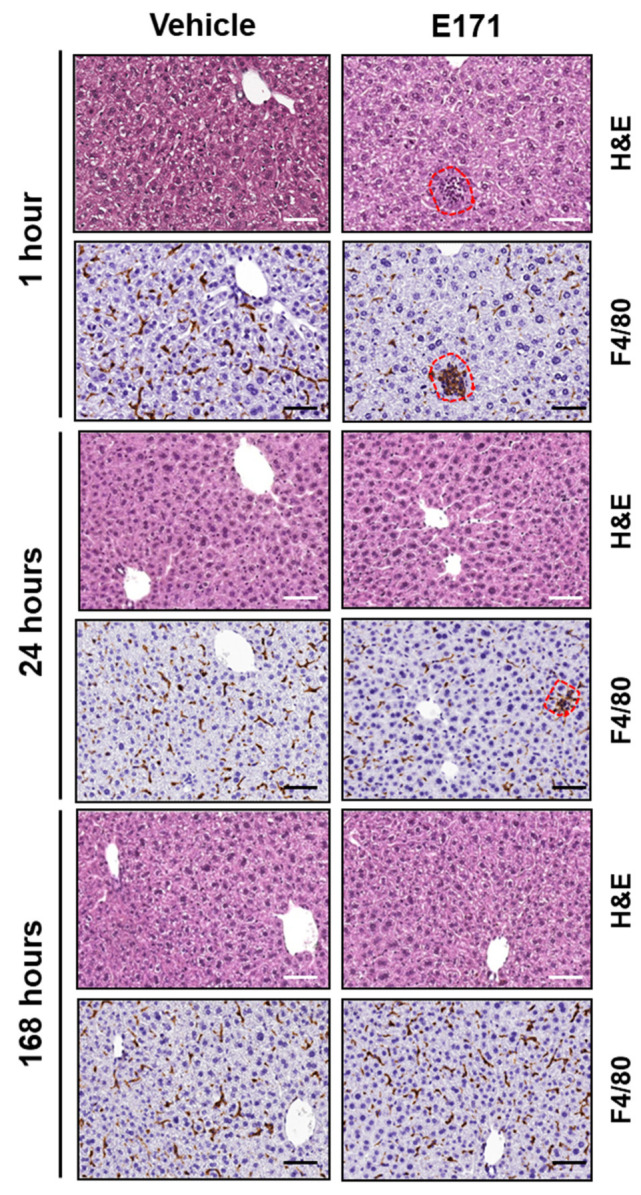
Pathological examination of livers of mice treated with E171. Representative images of liver hematoxylin–eosin (HE) and F4/80 immunostaining of mice injected with either vehicle or E171 and euthanized 1, 24, or 168 h after injection. Small microgranulomas were detected at 1 h and 24 h after E171 injection, but not at 168 h, indicative of low-grade transient inflammation due to E171. Scale bars = 50 μm.

To gain information on our hypothesis, groups of E171- and vehicle-treated mice were euthanized 1, 24, and 168 h after injection for a pathological evaluation of liver and lungs. Initially, livers were examined either by histopathological analysis (HE staining) or by immunostaining with F4/80 antibody, a marker of tissue-resident macrophages and monocytes. As expected, there were no marked signs of inflammation, steatosis, necrosis, or other tissue alterations in the vehicle control mice. Meanwhile, E171-treated mice exhibited small microgranulomas after only one hour (Figure 6, red dashed lines). These changes became less pronounced 24 h after E171 injection, and had completely disappeared 6 days later. These results suggest that TiO_2_ NPs (albeit in small amounts) can accumulate in liver parenchyma close to the F4/80^+^ resident intravascular Kupffer cells and/or the myeloid cells recruited from the circulation (Figure 4D). In order to eliminate NPs from the hepatic tissue, myeloid populations in the liver increased and formed transient microgranulomas, similar to the mice fed with TiO_2_ NPs and E171 [32,45]. ICP-MS data in Figure 3 indicate that, after E171 injection, Ti levels in the liver peaked within the first 24 h and returned to basal levels after 7 days. This suggests that the TiO_2_ NPs accumulated in the liver can be efficiently cleared out, causing a low and transient toxicity with no signs of chronic hepatic disease (see Figure 5). 

**Figure 7:**
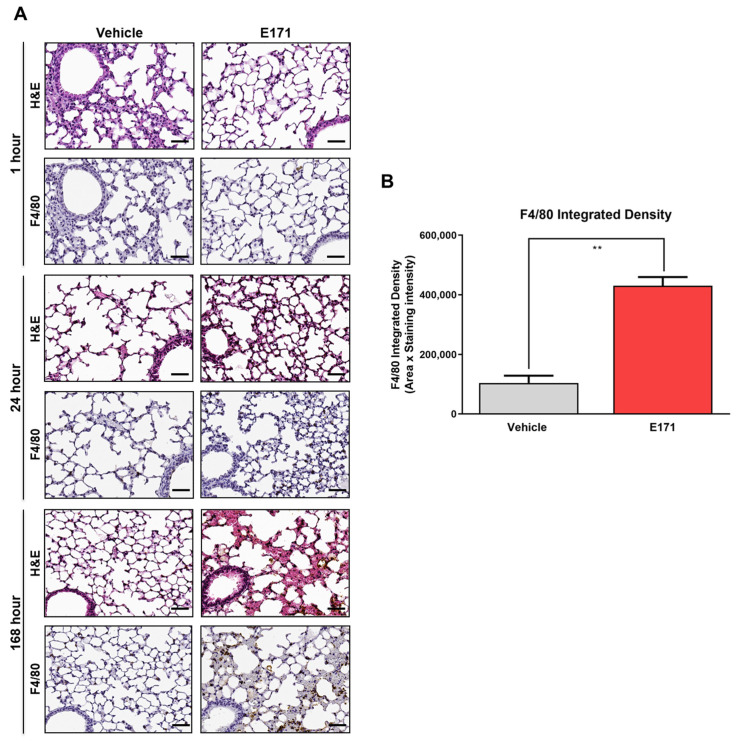
Pathological examination of the lungs of E171-injected mice. (**A**) Representative images of hematoxylin–eosin (HE) and F4/80 immunostaining of lung sections from mice treated with either vehicle or E171 and euthanized 1, 24, or 168 h after injection. (**B**) F4/80 immunoreactivity of slices from mice euthanized 168 h after treatment. Values are mean ± SEM (N = 5); ** *p* < 0.01 vs. vehicle, according to unpaired Student’s *t*-test. Scale bars = 50 μm.

To determine possible pulmonary morphological alterations induced by E171, at least 7 days after injection, the lungs (the organ with the highest Ti accumulation) of E171-injected mice (euthanized after 1, 24, or 168 h) were examined by histopathological analysis. To verify whether Ti accumulation can lead to direct modification of lung parenchyma, F4/80 antibody was used for immunostaining to detect macrophage accumulation. Contrary to what was observed in the liver, interstitial inflammation with F4/80^+^ cell recruitment was weak at the earliest time points and increased with Ti accumulation over time. While at one hour after E171 injection there was no evidence of lung parenchyma F4/80 staining—at 24 h, morphological alterations in lung tissues were observed. These were further enhanced in sections from animals euthanized 168 h after injection (Figure 7A). At the same time point, lungs of E171-treated mice displayed significantly more interstitial immunoreactivity in the alveoli compared with those injected with the control vehicle (Figure 7B). This progressive change was very likely due to an increased accumulation of E171 NPs in the lungs (see Figure 3). 

## 4. Discussion

TiO_2_ is a nanomaterial used in many fields, from environmental protection to agriculture, to food and cosmetic industries. Unfortunately, it may have potential side effects for humans due to the inevitable increase in exposure and various routes of entry (skin, respiratory tract, ingestion, etc.) [46]. In our work, we focused our attention to the potential impact of E171—the food-grade form of TiO_2_ widely used in the food industry—to estimate parameters such as biodistribution, accumulation, and toxicity in invertebrate and vertebrate animals.

Like other types of TiO_2_ NPs [34,35,36], E171 can be absorbed by *C. elegans*, passing biological barriers and exerting acute toxicological effects. Of particular concern is its effects on the reproduction and development of worms, which confirms the ability of TiO_2_ NPs to cause damage that can be transmitted from one generation to another. Toxicity scores in *C. elegans* have repeatedly proven to be predictive of toxic parameters in rodents [37,47,48], indicating that preclinical studies conducted only on nematodes cannot replace experiments on mammals for hazard identification, however, they can offer important translational information.

Recent studies have found evidence of downstream events outside the gastrointestinal tract, sharing the same mechanism of absorption through capillaries and vessels, entering the circulatory system with subsequent systemic diffusion [32,49,50,51]. Therefore, in the present study, we investigated the potential effects of single systemic over-exposure to E171 in immunocompetent healthy mice. In our previous study [32], ICP-MS showed that the liver had poor ability to retain E171. Although TiO_2_ NPs, considered hard nanomaterials, have a good tropism towards the liver [52] and can be internalized in the cells of the reticular endothelial system, the amount of hepatic Ti after intravenous administration of E171 was several times lower compared to that measured in previous studies [32,53]. 

Surprisingly, compared to gold NP kinetics [54], ICP-MS measurements showed a relatively low uptake of TiO_2_ NPs from the liver, with a complete disappearance after 7 days. This transient phenomenon seems to rely on the efficient uptake by hepatic macrophages. However, despite this minimal accumulation, transient histological changes were observed in liver parenchyma as early as one hour after E171 injection.

The lower absorptive capacity of hepatic components might indicate a greater filtering action from the spleen, or a rapid clearance and excretion by kidneys, which would support the safe use of these materials. However, renal levels of TiO_2_, as early as the first hour, did not seem to indicate that NPs were rapidly excreted in urine, and the very moderate accumulation in the spleen indicated a low uptake by red pulp macrophages [55].

Unexpectedly, there was a progressive and increasing accumulation of TiO_2_ in lungs of mice given a single intravenous dose of E171. The lack of further time points makes it difficult to establish whether day 7 is the peak of lung accumulation. However, this strange behavior obviously cannot be explained by the hypothesis that circulating NPs remain in the bloodstream for 7 days, given the ICP-MS plasma results. Therefore, it is possible that they somehow remain trapped in the endothelium where, from day one to day seven, the NPs are gradually released and taken up by lungs or are engulfed by circulating monocytes, as shown by the significantly higher number of monocytes in the blood of E171-treated animals 12 h after treatment. 

A possible explanation for why NPs in the 35 to 150 nm range are taken up by lungs—rather than by macrophages residing in the spleen and liver—is the irrelatively rapid aggregation in circulation [56]. Size plays a role in the organo-specific biodistribution of circulating particles. In general, spherical or spheroidal particles smaller than 10 nm are efficiently retained by renal tubules and then excreted in the urine, while particles from 500 nm to 1 µm remain trapped in lungs [57]. NPs from 10 to 500 nm generally accumulate efficiently in the liver and spleen at different rates, depending on surface area, charge, and coating. This assumption holds for stable NPs specifically synthesized and coated using surfactants to maintain a high degree of monodispersity in biological fluids. However, this is not the case for E171, where there is no precise surface functionalization, and they are not colloidally stable in a serum-containing medium (Figure 2). It is therefore likely that aggregates larger than 500 nm are formed in a gradual way, and that this “size transformation” may lead to concomitant release from the liver, while increasing accumulation in the lungs. The lack of colloidal stability after entering the bloodstream seems to be experimentally confirmed by TEM analyses of E171 after serum incubation. Our results point to an increase in the diameter of single particles and a lack of homogeneity in the shape and outer surface of NPs exposed to serum proteins— eventually leading to progressive aggregation and resulting in spurious microparticles [40]. Chen et al. [58] reported the formation of blood clots in the lungs of mice—treated by intraperitoneal injection with TiO_2_ NPs—due to the deposition of NPs in the blood vessels. As the levels of circulating hematopoietic cells were only taken into consideration 12 h after injection, the total number of platelets remained the same. However, it is possible that after a longer period, the formation of the blood clots caused a significant difference in the platelet levels, suggesting a platelet activation [59].

It is therefore possible that this unexpected increase in lung Ti levels is due to the formation of NP microaggregates, rather than an active mechanism of endothelial transcytosis or macrophage uptake. Alternatively, it is tempting to speculate that the Kupffer cells in the liver, along with the recruited monocytes/macrophages (after a first wave of hepatic uptake), die and release TiO_2_ into circulation in small amounts, secondarily aggregating in lungs—as indicated by the different kinetics of accumulation in these two organs. Consistent with this hypothesis, our recent study [54] demonstrated the strong influence of geometry on the biodistribution of gold NPs. Interestingly, neither size nor shape resulted in substantial toxicity in the treated mice. When NPs agglomerate, their initial size and shape loses importance [60]. This is an extremely important indication that it is not just how much material is accumulated, but where it accumulates in the parenchyma. The evidence of specific, well-limited foci of inflammation in the liver—despite such a small accumulation—strongly suggests the presence of microinfarcts in the sinusoids, due to being clogged with aggregated E171 NPs. The efficiency of the immune system to clear the debris of dying cells and the entrapped E171, is clear, as are the transient effects of this process. This may be responsible for both the disappearance of titanium between day one and day seven, as well as the absence of infiltrates in the liver. 

This study offers further information of the potential risks of exposure to TiO_2_ NPs in food products. Expanding on our recent work on mice [33], we examined the effects of acute exposure on nematodes (*C. elegans*) and found that E171 activated important pathological mechanisms—despite differences in the route of administration and morpho-functional differences between the two species. Therefore, this study not only reinforces the reliability of preliminary tests using *C. elegans* for bio–nano interaction studies, but also gives us the chance to focus on the fate of food-grade TiO_2_ NPs after internal absorption in mice.

The unexpected tropism towards lungs—with histopathological changes in animals treated systemically with E171—can plausibly be explained by the progressive loss of colloidal stability. This leads to the aggregation and entrapment of micrometric particles in the pulmonary microcirculation, preventing uptake through hepatic sinusoids. Although further studies may be needed to investigate specific modes of administration and chronic exposure—this analysis offers useful insights in the evaluation of the physicochemical parameters of NPs. The results support the recent decision to ban E171 in Europe and limit the use of TiO_2_ NPs to avoid any impact on human and environmental health.

## Data Availability

The data presented in this study are available upon request from the corresponding author.
